# News and Notes

**Published:** 1902-12

**Authors:** 


### NEWS AND NOTES.

(We are indebted to the *Medical Age* for many of these notes.)

---

THE N. O. Polyclinic opened for its sixteenth annual session on November 3, 1902. Up to date the class is large and the prospects are brilliant for a very successful term.

---

TWO cases of leprosy in Iowa have been reported to the State Board of Health.

---

AN anti-mosquito campaign in Corsica has brought about a great improvement as regards the prevalence of malarial fevers there.

---

THE Massachusetts State Board of Health states that in the past seven years nearly 11,000 lives were saved by the use of antitoxin.

---

DR. A. S. ASHMEAD, of New York, advocates an international leper treaty between the United States and Japan, owing to the fact that there are nearly 100,000 lepers in Japan.

---

THE Southern Surgical and Gynecological Association held its fifteenth annual meeting, under the presidency of Dr. W. E. B. Davis, of Birmingham, Ala., in Cincinnati on November 11, 12, and 13, 1902.

---

THE nineteenth annual meeting of the Interstate Medical Association of Mississippi, Arkansas, and Tennessee was held at Memphis, Tenn., November 11, 12, and 13, under the presidency of Dr. J. B. McElroy.

THE third annual meeting of the American Roentgen Ray Society will be held in Chicago, December 10 and 11. The sessions of the society, as well as the exhibits, will be held in the Sherman House, Randolph and Clark streets.

---

ACCORDING to the *Lancet* there are 1211 medical men of all nationalities in Egypt, of whom 604 are Europeans, 85 hold a Persian or Turkish diploma from Teheran or Constantinople, 45 are natives with a European diploma, and the remaining 477 are natives educated at Cairo in the one Egyptian medical school. The number of Greek physicians shows a steady increase.

---

PROF. FRIEDRICH KRAUS, of Graz, Austria, who has been appointed to succeed the late Professor Gerhardt in the chair of clinical medicine at Berlin, is a native of Bohemia and is forty-four years of age. He studied at Prague and Vienna, acted as assistant to Professor Kahler, and in 1893 was appointed professor extraordinary at Vienna University, receiving the appointment of a full professorship at Graz the following year.

---

THE American Committee of the XIVth International Congress of Medicine announces that blank forms of application addressed to the Bureau des Logements in Madrid, forms of applivation for those who desire to read papers at the Congress, as well as other literature bearing on the subject may be obtained upon request from the secretary of the committee, John H. Huddleston, M.D., 186 West 85th street, New York City.

---

THE Berlin Academy of Sciences has announced (*Popular Science Monthly*) that its academic prize of 5000 marks will be awarded in 1904 for an investigation of the cathode rays, and in 1905 for an investigation of the theory of functions of several variables which admit of linear substitution. The income of the Cothemus legacy

($2,000) for 1904 will be awarded for investigations of new varieties of grain. The papers may be written in English, and must be presented, without the name of the author, to the Bureau of the Academy, Universitatstrasse 8, Berlin.

---

CAPTAIN POTTER, of the United State steamship *Ranger*, writes from Panama, September 29, that the health conditions of the isthmus are very unsatisfactory, both yellow fever and dysentery prevailing among the Colombian troops stationed on the isthmus. The steamship *Ecuador*, which reached Panama October 12, was held five miles out in the bay, because there was a case of yellow fever on board. The patient, according to the *Philadelphia Medical Journal*, died soon after the vessel was placed in quarantine, where she was held eight days. No cases of yellow fever are reported in the city of Panama.

---

W. S. SACKETT, a graduate of the University of Chicago, says the *Philadelphia Medical Journal*, has recently undertaken a number of investigations upon the preference of mosquitoes for certain colors. He has found, so far, that red and black attract great quantities of mosquitoes. This, however, seems to contradict the old notion that a piece of red ribbon across the window-sill is more effective in keeping out mosquitoes than are screens. In his investigations he has used the Culex mosquito. It is remarkable that no mosquito alights upon yellow. He therefore draws the conclusion that houses should be painted yellow, and that yellow clothes should be worn, to keep free from mosquitoes.

---

UNDER date of July 18, 1902, Consul Charles V. Herdliska, of Callao, Peru, in reply to an inquiry regarding the chances of success for an American physician in Lima, writes as follows: "Before a physician can enter upon the practice of his profession in

Peru he must pass a State examination upon medicine, conducted in the Spanish language. Upon being found qualified a certificate is issued which entitles him to practice his profession in any part of the Republic. The opportunities for American physicians would seem to be good. Both Lima and Callao contain quite a large American and English colony, and the Peruvians themselves appear to have great faith in the American, English, German, and French Physicians and surgeons, on account of the advanced state of medical science in those countries."—*Medical Record.*

---

DR. HALDANE, of Liverpool (*American Medicine*), believes that carbonic oxide, blown through all parts of the ship before the cargo is broken, destroys all rats on board, and thus prevents plague infection. The carbonic oxide is generated by blowing air through a bed of incandescent coke or anthracite in a special furnace. The result, known as producer gas, contains three parts of carbonic oxide to seven of nitrogen. When a vessel comes into dock the necessary apparatus is brought alongside, and the poisonous air is blown through the hold, entering through a ventilator in the upper part and passing out through an opening of the lower part into the next hold. The tarpaulins covering the hatches are not removed until the operation is completed. The poisonous air is then blown out with pure air, and the hatches are opened. To determine whether any poison remains, a small bird or a mouse is lowered into the different holds. Haldine's experiments showed that all rats were killed in nine minutes.

